# Cortistatin reduces atherosclerosis in hyperlipidemic ApoE-deficient mice and the formation of foam cells

**DOI:** 10.1038/srep46444

**Published:** 2017-04-13

**Authors:** Virginia Delgado-Maroto, Raquel Benitez, Irene Forte-Lago, Maria Morell, Elena Maganto-Garcia, Luciana Souza-Moreira, Francisco O’Valle, Mario Duran-Prado, Andrew H. Lichtman, Elena Gonzalez-Rey, Mario Delgado

**Affiliations:** 1 Institute of Parasitology and Biomedicine Lopez-Neyra, CSIC, Granada, Spain; 2 Department of Pathology, Brigham and Women’s Hospital, Harvard Medical School, Boston, USA; 3 Department of Pathology, School of Medicine, University of Granada, Granada, Spain; 4 Medical Sciences, University of Castilla-La Mancha, Ciudad Real, Spain

## Abstract

Atherosclerosis is a chronic inflammatory cardiovascular disease that is responsible of high mortality worldwide. Evidence indicates that maladaptive autoimmune responses in the arterial wall play critical roles in the process of atherosclerosis. Cortistatin is a neuropeptide expressed in the vascular system and atherosclerotic plaques that regulates vascular calcification and neointimal formation, and inhibits inflammation in different experimental models of autoimmune diseases. Its role in inflammatory cardiovascular disorders is largely unexplored. The aim of this study is to investigate the potential therapeutic effects of cortistatin in two well-established preclinical models of atherosclerosis, and the molecular and cellular mechanisms involved. Systemic treatment with cortistatin reduced the number and size of atherosclerotic plaques in carotid artery, heart, aortic arch and aorta in acute and chronic atherosclerosis induced in apolipoprotein E-deficient mice fed a high-lipid diet. This effect was exerted at multiple levels. Cortistatin reduced Th1/Th17-driven inflammatory responses and increased regulatory T cells in atherosclerotic arteries and lymphoid organs. Moreover, cortistatin reduced the capacity of endothelial cells to bind and recruit immune cells to the plaque and impaired the formation of foam cells by enhancing cholesterol efflux from macrophages. Cortistatin emerges as a new candidate for the treatment of the clinical manifestations of atherosclerosis.

Cardiovascular disease has become the most frequent cause of death globally. Although many diseases affect the cardiovascular system, myocardial infarction and ischemic stroke caused by atherosclerosis dominate mortality and morbidity statistics worldwide. Far to the traditional view of atherosclerosis resulting from a passive build up of cholesterol in the artery wall, there is now much evidence that chronic inflammation and autoimmune mechanisms are critically involved in its pathogenesis, especially during progression phase and during the onset of vulnerable plaques, as reflected by the large number of monocyte-derived macrophages and T lymphocytes within the atherosclerotic plaque^1^,^2^,^3^. These findings raise the possibility that immunomodulatory therapeutic approaches may be successful in this cardiovascular disease, alone or in addition to lipid-lowering therapy.

Cortistatin is a neuropeptide initially discovered in brain cortex and hypothalamus based in its inhibitory neuronal activities^4^ that recently emerged as a pleiotropic immunomodulatory factor^5^. Due to its remarkable sequential/structural resemblances with somatostatin, cortistatin exerts many somatostatin-like functions, especially concerning hormonal and neuronal regulation^6^,^7^. However, cortistatin plays unique functions in brain and immune system^4^,^5^,^6^,^7^,^8^, which are related to its capacity to bind to receptors other than those somatostatin, mainly the ghrelin-receptor^7^,^9^,^10^. Although its role in the cardiovascular system is largely unknown, recent evidence invites to the study of the effect of cortistatin in inflammatory cardiovascular diseases. First, the cardiovascular system produces cortistatin, especially in response to injury. Thus, heart, arterial endothelium and arterial smooth muscle cells (SMCs) express cortistatin and its receptors^10^,^11^,^12^. Moreover, cortistatin is expressed in human atherosclerotic plaques, specifically in SMCs and infiltrating inflammatory cells and its expression increases in complex plaques^11^. Cortistatin expression also augmented in mouse arteries subjected to blood-flow alterations, in plasma of patients with coronary artery lesions and in hearts of mice with autoimmune myocarditis^11^,^13^,^14^. Second, treatment with cortistatin improves vasculopathies associated with vascular remodelling and reduces experimental arterial stenosis and vascular calcification^11^,^15^. In agreement, cortistatin-deficient mice responded to neointimal lesion with exacerbated vascular responses^11^. Finally, cortistatin regulates both innate and adaptive immune responses in many inflammatory and autoimmune disorders, including experimental autoimmune myocarditis, collagen-induced arthritis, experimental autoimmune encephalomyelitis and inflammatory bowel disease^8^,^14^,^16^,^17^,^18^.

Therefore, here we investigated the effect of cortistatin in two experimental models of acute and chronic atherosclerosis induced in apolipoprotein E-deficient (apoE^−/−^) mice fed a high-lipid Western diet, as well as the potential cellular and molecular mechanisms involved.

## Results

### Cortistatin protects from atherosclerosis in partially ligated carotid artery of hyperlipidemic mice by impairing Th1/Th17-driven inflammatory responses

**Figure 1:**
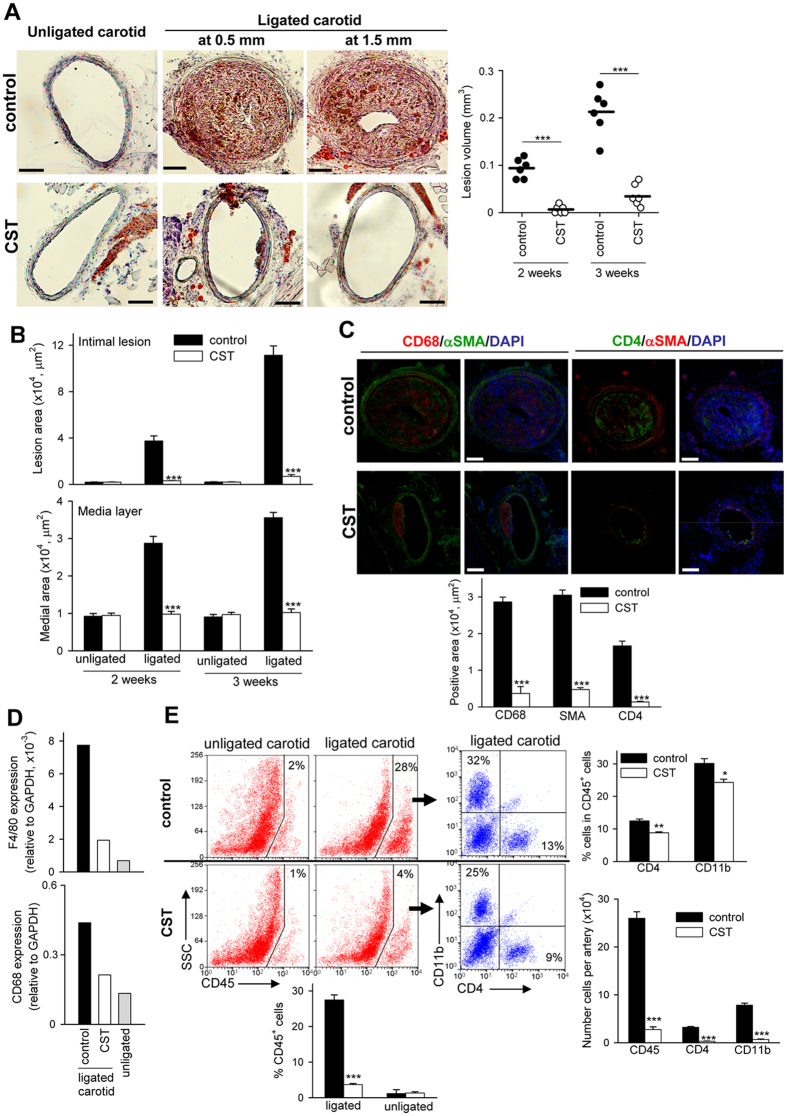
Cortistatin protects from acute atherosclerosis in carotid artery in hyperlipidemic mice. ApoE^−/−^ mice subjected to partial ligation of left carotid artery and fed a high-fat diet were intraperitoneally treated with PBS (control) or with cortistatin (CST) every other day starting one day after ligation. Contralateral unligated carotid and ligated carotid were collected two and three weeks after ligation. (**A**) The volume of atherosclerotic lesions was determined by morphometry in carotid sections isolated 3 weeks after ligation. Images correspond to Oil red O-stained cryosections obtained at 0.5 or 1.5 mm proximal to carotid bifurcation. Each symbol in right panel represents 1 mouse, and horizontal lines correspond to the mean value of each experimental group. (**B**) Areas of intimal lesions and media layers were measured by morphometry in sections stained with hematoxylin-eosin or Oil red O. Supplementary Fig. S1 shows intima/media ratio values. n = 14 mice per group, performed in three experiments. (**C**) Immunofluorescence analysis of CD68^+^ macrophages, smooth muscle α-actin^+^ (SMA) cells and CD4^+^ lymphocytes in carotid sections isolated 3 weeks after ligation. Nuclei were DAPI-counterstained. Supplementary Fig. S1 shows immunostaining negative controls. n = 6 mice per group, performed in two experiments. (**D**) The presence of macrophages was confirmed by qPCR analysis of CD68 and F4/80 mRNA expression in carotids isolated 3 weeks after ligation. n = 4 mice per group, pooled in two samples. (**E**) Flow cytometric analysis of cell infiltrates isolated from carotids 3 weeks after ligation. The percentages of CD45^+^ leukocytes in whole carotid cell isolates, and of CD11b^+^ monocytes and CD4^+^ lymphocytes in gated CD45^+^ cells were determined. The numbers of total CD45^+^, CD11b^+^ and CD4^+^ cells per carotid artery are shown. n = 6 mice per group, performed in two experiments. Scale bars: 100 μm. *p < 0.05, **p < 0.001, ***p < 0.0001 *vs* control.

We investigated the potential therapeutic effect of cortistatin in two models of atherosclerosis in genetically susceptible apoE^−/−^ mice fed a high-lipid diet. In the first model, we ligated three of the four caudal branches of the left common carotid artery (Supplementary Fig. S1A) and then fed animals with a Western diet for 3 weeks. This partial ligation causes disturbed blood-flow with low and oscillatory shear stress and accelerated atherosclerosis in the carotid artery of hyperlipidemic mice^19^. In comparison to contralateral unligated artery, ligated carotid developed severe atherosclerotic plaques that progressively occupied the vascular lumen (Fig. 1A and B, Supplementary Fig. S1). The plaques were characterized by the predominant presence of cells stained with Oil Red O, a marker of lipid accumulation, probably corresponding to lipid-loaded macrophages (Fig. 1A). Studies of immunofluorescence confirmed the presence of infiltrating CD68^+^ macrophages, as well as of CD4^+^ T cells and α-actin^+^ SMCs, in the carotid plaques (Fig. 1C). Moreover, in comparison to unligated carotid, ligated carotid showed increased gene expression of the macrophage markers CD68 and F4/80 (assayed by qPCR, Fig. 1D), and higher presence of CD45^+^ leukocytes, including CD11b^+^ macrophages and CD4 T lymphocytes (analyzed by flow cytometry, Fig. 1E). Systemic administration of cortistatin three times per week, during two weeks resulted in a strong reduction in the formation of atherosclerotic plaques in the ligated carotid (Fig. 1A, Supplementary Fig. S1). Moreover, cortistatin treatment decreased the presence of macrophages, CD4 T cells and SMCs in the plaque (Fig. 1C and D). Interestingly, cortistatin also impaired the increase observed in carotid medial layer area, which is a consequence of vascular remodelling that occurs after artery ligation (Fig. 1B, Supplementary Fig. S1).

**Figure 2:**
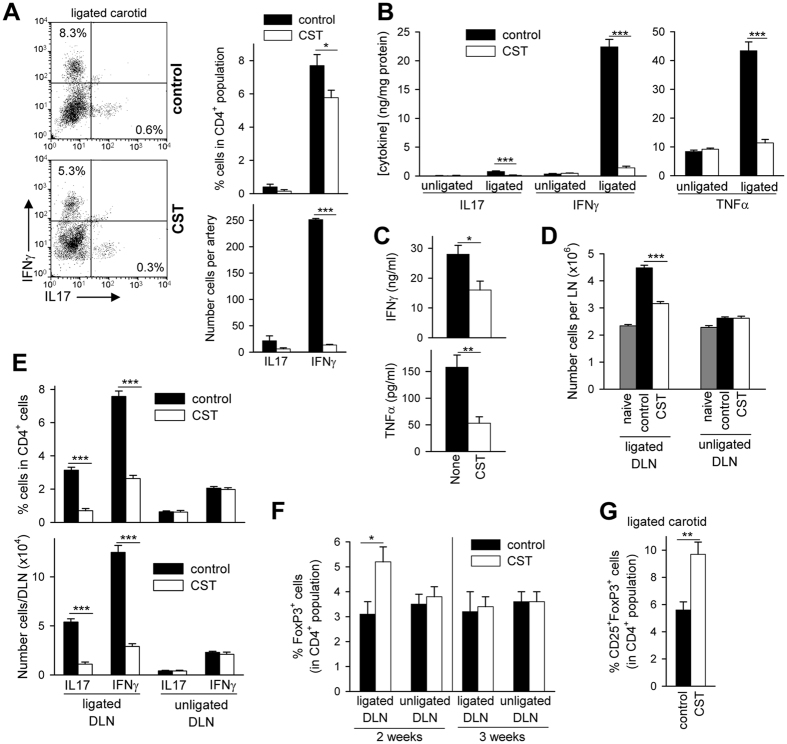
Cortistatin downregulates local and peripheral inflammatory and T-cell responses in acute atherosclerosis. ApoE^−/−^ mice subjected to partial ligation of left carotid artery and fed a high-fat diet were treated with PBS (control) or with cortistatin (CST) every two days, starting one day after ligation. (**A**) The presence of IL17- and IFNγ-producing T cells in carotids was determined by flow cytometric analysis of cells isolated from carotids obtained 3 weeks after ligation. Plots correspond to intracellular cytokine staining in gated CD4^+^ cells. n = 6 mice per group. (**B**) Cytokine levels were determined in protein extracts isolated from carotid arteries 3 weeks after ligation and expressed as ng of cytokine per mg of protein. n = 5 mice per group. (**C**) Cytokine production by ligated carotids isolated from untreated control mice 3 weeks after ligation and *ex vivo* activated in the absence (none) or presence of cortistatin. n = 6 mice per group, performed in two independent experiments. **p < 0.0001 *vs* control. (**D**) Total number of cells in draining lymph nodes (DLNs) of ligated carotid and of contralateral unligated carotid isolated 2 weeks after ligation. Naïve apoE^−/−^ mice fed a normal chow diet were used as reference. Supplementary Fig. S2 shows values determined in non-draining mesenteric lymph nodes and spleen. n = 5 mice per group. (**E**) Number and percentages of IFNγ-producing CD4 T cells and IL-17-producing CD4 T cells in DLNs of ligated and unligated carotid arteries isolated 2 weeks after ligation were determined by flow cytometric analysis. n = 5 mice per group. Supplementary Fig. S2 shows values obtained in DLNs and spleens isolated 3 weeks after ligation and the percentage of CD4^+^ cells in DLNs and spleens. (**F**) Flow cytometric analysis of FoxP3^+^ cells in gated CD4^+^ lymphocytes of DLNs of unligated and ligated carotid arteries. n = 5 mice per group. (**G**) Percentage of CD25^+^ FoxP3^+^ cells in gated CD4^+^ lymphocytes determined by flow cytometry in cell suspensions isolated from ligated carotids 3 weeks after ligation. n = 6 mice per group. *p < 0.05; **p < 0.001; ***p < 0.0001 *vs* control.

Because atherogenic Th1 and Th17 cells play a major role in development and progression of the atherosclerotic plaque^1^,^2^, we evaluated the effect of cortistatin treatment in the presence of these pathogenic cells in plaques and in lymphoid organs. As expected, flow cytometric analysis of single-cell isolates of ligated carotid of untreated mice showed that plaque-infiltrating CD4 T cells mainly included IFNγ-producing Th1 cells, and some IL-17-producing Th17 cells (Fig. 2A). Determination of cytokine contents in protein extracts of ligated carotids bearing atherosclerotic plaques confirmed the increased expression of inflammatory cytokines linked to development of atherosclerosis^20^, including TNFα and IFNγ (Fig. 2B). Moreover, *ex vivo* activation of ligated carotid resulted in high secretion of IFNγ and IL-17 (Supplementary Fig. S2A). In contrast, arteries isolated from hyperlipidemic mice that were treated with cortistatin showed low numbers of IFNγ- and IL-17-producing CD4 T cells (Fig. 2A) and produced low levels of inflammatory cytokines (Fig. 2B, Supplementary Fig. S2A). Moreover, the addition of cortistatin to cultures of ligated carotid arteries diminished the production of TNFα and IFNγ, suggesting a direct suppressive effect of the neuropeptide in atherosclerotic plaques (Fig. 2C).

On the other hand, we observed that the number of total cells in draining lymph nodes (DLNs) of the ligated carotid increased with respect to the corresponding DLNs isolated in the contralateral side of the same animal (DLNs of the unligated artery) or DLNs of naïve apoE^−/−^ mice fed a normal chow diet (Fig. 2D), or with respect to spleen or mesenteric lymph nodes (non-DLNs) of apoE^−/−^ mice fed a Western diet during 3 weeks (Supplementary Fig. S2B), pointing out to a major immune response occurring in DLNs of ligated carotids. Analysis of IFNγ- and IL-17-producing CD4 cells by flow cytometry indicated a moderate increase in these atherogenic T cell populations in DLNs of ligated carotid, but not in contralateral DLNs or in spleen (Fig. 2D, Supplementary Fig. S2C–E). Treatment with cortistatin decreased the numbers of total cells in DLNs, especially of both Th1 and Th17 cells (Fig. 2D and E, Supplementary Fig. S2B–E). In contrast, the percentage of FoxP3^+^ regulatory T cells (Treg) in the CD4 population of DLNs significantly increased (p = 0.02) with cortistatin treatment 14 days after ligation (Fig. 2F), which correlated with an elevation of this cell population in the ligated carotid artery (Fig. 2G). Although cortistatin did not significantly affect the percentage of Treg cells in DLNs at later time points (3 weeks after ligation, Fig. 2F), it restored the ratios between Th1 cells and Treg (Th1/Treg ratio of 0.5 for naïve apoE^−/−^ mice, Th1/Treg ratio of 1.8 for untreated atherosclerotic mice and Th1/Treg ratio of 0.6 for cortistatin-treated atherosclerotic mice). Therefore, these findings indicate that treatment with cortistatin protects from acute atherosclerosis in carotid artery and impairs Th1 and Th17-driven inflammatory responses at the vessels and DLNs.

### Cortistatin decreases the formation of plaques in a model of chronic atherosclerosis

**Figure 3:**
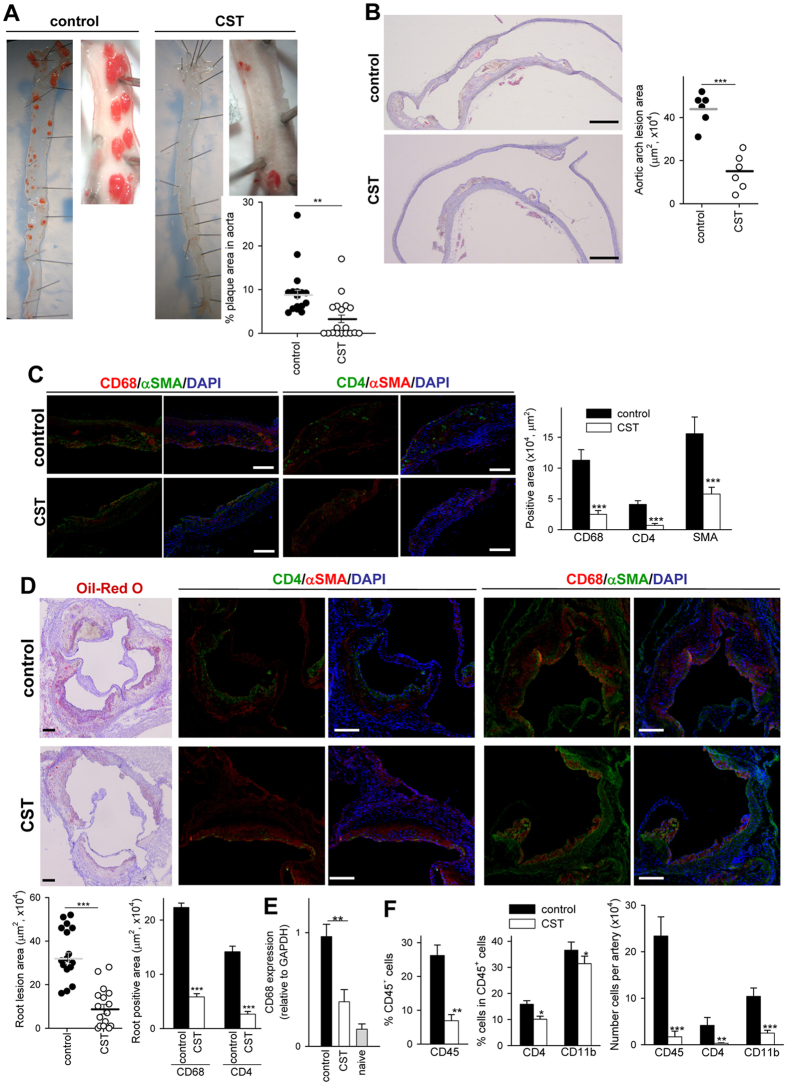
Cortistatin protects from hyperlipidemic diet-induced chronic atherosclerosis. ApoE^−/−^ mice fed a high-fat diet during 16 weeks were intraperitoneally treated with PBS (control) or cortistatin (CST) three times per week, starting one week after initiation of diet. (**A**) Images of descending aorta stained with Sudan IV and quantification of percentage of aorta area occupied by plaque. Each symbol corresponds to 1 mouse. Horizontal lines are the mean and vertical lines represent s.e.m. for each experimental group. (**B**) Images of longitudinal sections of aortic arch stained with Oil Red O and quantification of atherosclerotic lesion area. Scale bars: 500 μm. Images and quantification of cross-sections of aortic arch are shown in Supplementary Fig. S3. (**C**) Images of sections of aortic arch immunostained for infiltrating CD68^+^ macrophages and CD4^+^ T cells and for αSMA^+^ smooth muscle cells. Quantification of positive areas for each cell type is shown (n = 6 mice/group). Scale bars: 100 μm. (**D**) Images of sections of aortic root sinus stained with Oil Red O and immunostained for CD68, CD4 and αSMA. Quantification of atherosclerotic lesion area (n = 16 mice/group) and of positive area for each cell type (n = 6 mice/group) is shown. Scale bars: 250 μm. (**E**) mRNA expression of CD68 in aortic arch (n = 5 mice/group). Naïve C57BL/6 mice fed a normal chow diet were used as reference control. (**F**) Flow cytometric analysis of cell infiltrates isolated from aortic artery. The percentages of CD45^+^ leukocytes in whole cell isolates, and of CD11b^+^ monocytes and CD4^+^ lymphocytes in gated CD45^+^ cells, as well as, the number of CD45^+^, CD11b^+^ and CD4^+^ cells per aortic arch were determined. n = 8 mice per group, performed in two experiments. *p < 0.05, **p < 0.001, ***p < 0.0001 *vs.* control.

Next, we investigated potential protective effects of cortistatin in a model of chronic atherosclerosis in apoE^−/−^ mice fed a high-lipid Western diet for 16 weeks. Systemic treatment with cortistatin, 3 times per week, reduced the number and size of atherosclerotic lesions in descending aorta, aortic sinus and aortic arch (Fig. 3A–C). Remarkably, 55% and 35% of cortistatin-treated mice did not show evidence of plaque formation in the descending aorta and aortic sinus, respectively, at the end of treatment. Atherosclerotic animals treated with cortistatin showed plaques in aortic sinus and aortic arch with lower numbers of leukocytes in general, and of macrophages and CD4 T cells in particular (Fig. 3B–D). Moreover, treatment with cortistatin reduced the presence of α-actin^+^ SMCs in the plaques of aortic arch and sinus (Fig. 3B and C) and diminished the medial muscle layer thickening in aortic arch (Supplementary Fig. S3).

**Figure 4:**
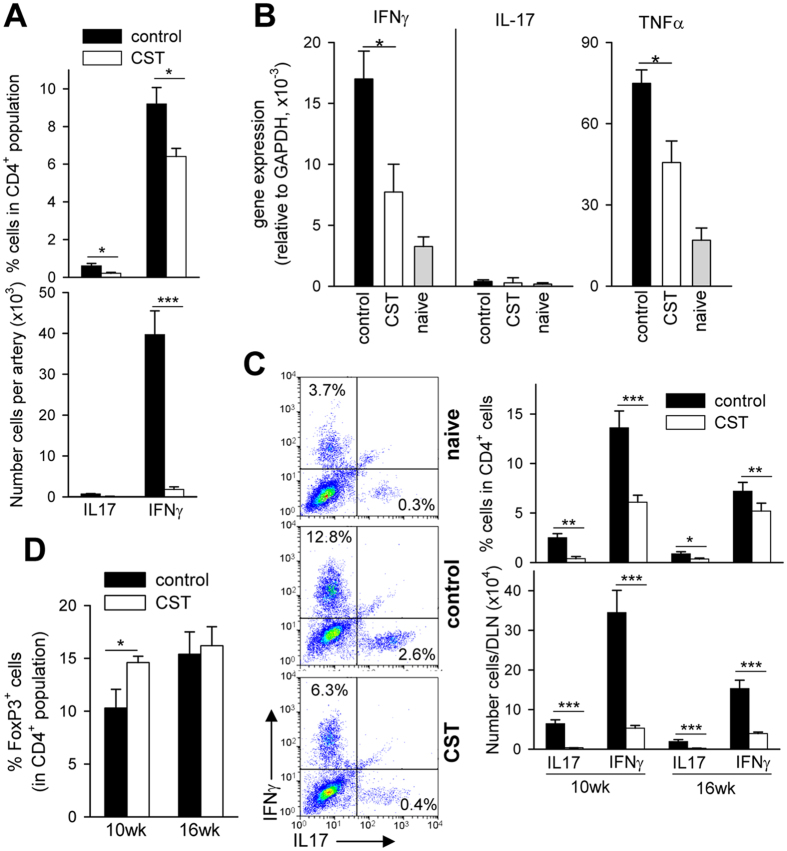
Cortistatin reduces the number of aortic and peripheral Th1 and Th17 cells in hyperlipidemic mice. ApoE^−/−^ mice fed a high-fat diet were treated with PBS (control) or cortistatin (CST) three times per week starting one week after initiation of diet. (**A**) The presence of IL17- and IFNγ-producing T cells in aorta was determined by flow cytometric analysis of cells isolated from aortic arch 16 weeks after initiation of diet. n = 8 mice per group. (**B**) Cytokine levels were determined by qPCR in mRNA isolated from aortic arch 16 weeks after initiation of diet. Naïve female C57Bl/6 (22 weeks old) mice fed a normal chow diet were used as reference. n = 5 mice/group. (**C**) Number and percentages of IFNγ-producing CD4 T cells and IL-17-producing CD4 T cells in DLNs of aorta isolated 10 or 16 weeks after initiation of diet were determined by flow cytometric analysis. n = 8 mice/group. Plots correspond to intracellular cytokine staining in gated CD4^+^ cells. Supplementary Fig. S5 shows values of total cell numbers and percentages of CD4^+^, IFNγ^+^ and IL-17^+^ cells in DLNs, spleen and non-draining mesenteric LN. (**D**) Percentage of CD25^+^FoxP3^+^ cells in gated CD4^+^ lymphocytes were determined by flow cytometry in DLNs of aorta isolated 10 or 16 weeks after initiation of diet. n = 8 mice per group. *p < 0.05; **p < 0.001; ***p < 0.0001 *vs* control.

Regarding the mechanisms involved in the protective effect of cortistatin in chronic atherosclerosis, we found that cortistatin treatment did not significantly change the plasma cholesterol concentrations in atherosclerotic mice (Supplementary Fig. S4). Other parameters including food intake, body weight or glucose levels were not affected by cortistatin treatment (Supplementary Fig. S4). As expected, aortic plaques of apoE^−/−^ mice fed a high-lipid diet showed many IFNγ-producing T cells, and a marginal presence of IL-17-producing CD4 cells (Fig. 4A), and high mRNA expression of IFNγ, and the inflammatory cytokine TNFα (Fig. 4B). Cardiac and aortic DLNs of hyperlipidemic mice showed higher cellularity and percentages of CD4 cells and of T cells expressing IL-17 and IFNγ than aortic DLNs of naive C57Bl/6 mice fed a normal diet and than non-DLNs of atherosclerotic mice (Fig. 4C, Supplementary Fig. S5A). In addition, spleens of apoE^−/−^ mice with chronic atherosclerosis showed increased numbers of total and Th1 cells in comparison to spleens of naïve C57Bl/6 mice fed a normal diet (Supplementary Fig. S5). Treatment with cortistatin normalized the number and percentages of Th1 and Th17 cell populations in aortic DLNs and spleen (Fig. 4C, Supplementary Fig. S5). At the same time, apoE^−/−^ mice treated with cortistatin showed elevated percentages of FoxP3^+^ Treg cells in DLNs at week 10, but not at week 16, in comparison to untreated mice (Fig. 4D). These findings support that downregulation of the atherogenic T cell-mediated responses during the effector phase of the disease could play a role in the protective effect of cortistatin in atherosclerosis.

### Cortistatin impairs the capacity of endothelial cells to bind immune cells

**Figure 5:**
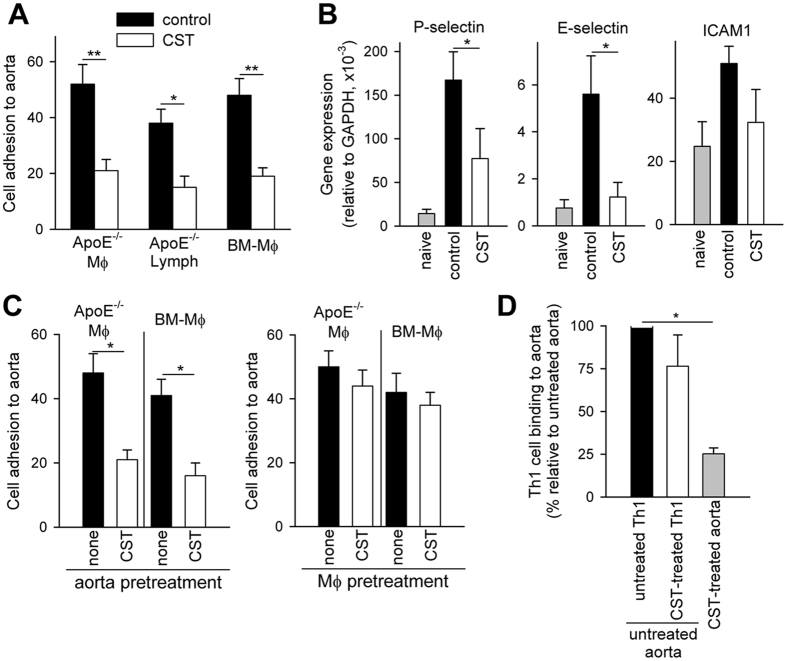
Cortistatin impairs binding of macrophages and lymphocytes to atherosclerotic aortas. (**A**) ApoE^−/−^ mice fed a high-fat diet were treated with PBS (control) or cortistatin (CST) three times per week starting one week after initiation of diet. Aortas were isolated 12 weeks after initiation of diet and assayed for *ex vivo* cell adhesion of peritoneal macrophages (apoE^−/−^ MΦ) or DLN lymphocytes isolated from untreated atherosclerotic apoE^−/−^ mice (at week 16) or of bone marrow-derived macrophages (BM-MΦ) isolated from naïve C57Bl/6 mice. n = 5 mice/group, performed in duplicates. (**B**) Gene expression of adhesion molecules was determined by qPCR in the RNA samples isolated from aortas isolated from PBS-treated (control) or cortistatin-treated atherosclerotic apoE^−/−^ mice 12 weeks after initiation of diet. Naïve C57Bl/6 mice fed a normal chow diet were used as reference. n = 5 mice/group. (**C**) Aortas isolated from untreated atherosclerotic apoE^−/−^ mice 12 weeks after initiation of diet were *ex vivo* pretreated with medium (none) or cortistatin and then assayed for binding of peritoneal macrophages (apoE^−/−^ MΦ) isolated from untreated atherosclerotic apoE^−/−^ mice (at week 16) or for binding of bone marrow-derived macrophages (BM-MΦ) isolated from naïve C57Bl/6 mice (*left panel*). Similarly, apoE^−/−^ MΦ or BM-MΦ were pretreated with medium (none) or with cortistatin before assaying their binding capacity to atherosclerotic aortas (*right panel*). (**D**) Th1 cells differentiated in the absence (untreated Th1) or presence of cortistatin (CST-treated Th1) were assayed for binding to aortas activated with inflammatory stimuli. Moreover, mouse aortas were preincubated with cortistatin (100 nM, grey column) before the addition of Th1 cells to culture. n = 3, in duplicates. *p < 0.05, **p < 0.001.

A crucial event in the atherogenic process is the binding of infiltrating cells with adhesion molecules expressed by activated endothelial cells of the plaque and their transmigration through the endothelial layer^1^. Therefore, we investigated the capacity of cortistatin to regulate these events by using different experimental approaches. We found that aortas obtained from cortistatin-treated atherosclerotic mice showed impaired capacity to bind macrophages and lymphocytes isolated from hyperlipidemic apoE^−/−^ mice or even bone marrow-derived macrophages isolated from naïve C57Bl/6 mice (Fig. 5A). In agreement, aortas isolated from cortistatin-treated apoE^−/−^ mice showed reduced expression of the adhesion molecules P-selectin, E-selectin and ICAM (Fig. 5B), suggesting that cortistatin could regulate the capacity of endothelial cells to bind immune cells. These results were partially confirmed using human vascular endothelial cells activated with oxidized low-density lipoprotein (oxLDL), a well-established *in vitro* atherosclerotic model of cell adhesion (Supplementary Fig. S6A and S6B). Moreover, *in vitro* treatment with cortistatin reduced the capacity of aortas obtained from atherosclerotic mice to recruit macrophages isolated from apoE^−/−^ or naïve C57Bl/6 mice (Fig. 5C, *left panel*). However, pretreatment of macrophages with cortistatin did not significantly affect their adhesion to atherosclerotic aortas (Fig. 5C, *right panel*). Similarly, treatment with cortistatin of mouse aortas activated with inflammatory stimuli reduced their capacity to bind Th1 cells (Fig. 5D). Again, the presence of cortistatin during the generation of DLN Th1 cells did not significantly affect their adhesion to activated aortas (Fig. 5D). Similarly, Th1 cells differentiated in the presence of cortistatin did not change their binding capacity to P-selectin and E-selectin chimeras at the flow rate occurring in aortic arch (Supplementary Fig. S6C). Moreover, cortistatin showed no significant effect on the migratory capacity of macrophages *in vivo* and *in vitro* (Supplementary Fig. S6D and S6E). These findings suggest that cortistatin may impair the capacity of endothelial cells to recruit monocytes and atherogenic T cells into the plaque.

### Cortistatin reduces the formation of foam cells by increasing the efflux of cholesterol

**Figure 6:**
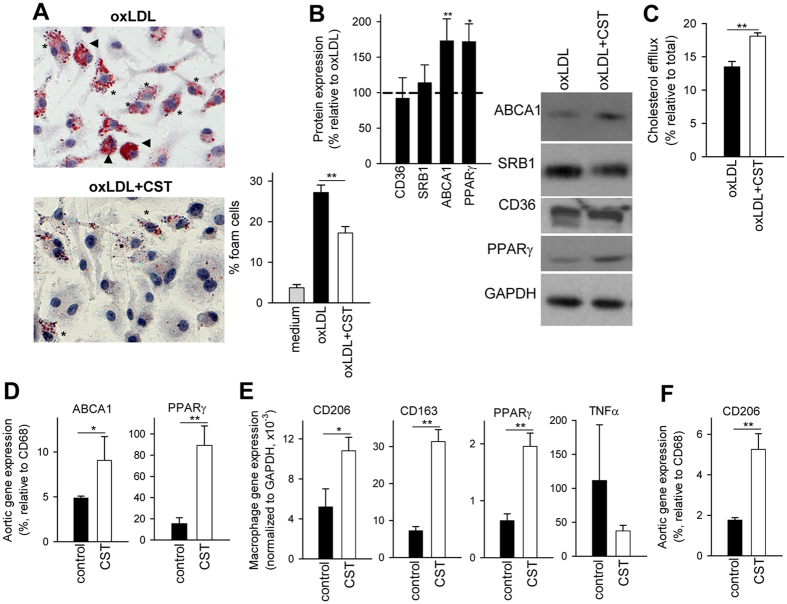
Cortistatin limits the formation of foam cells by promoting cholesterol efflux in macrophages. (**A**) Bone marrow-derived macrophages cultured in medium or stimulated with oxidized-LDL (oxLDL) in the absence or presence of cortistatin (CST, 100 nM) were stained with Oil Red O. The percentage of foam cells with mild (asterisk) and high (arrow head) content of lipid droplets was quantified by microscopy. Images are representative of four experiments performed in triplicate. (**B**) Expression of CD36, scavenger receptor-1 (SRB-1), ABCA1 and PPARγ by macrophages exposed to oxLDL in the absence or presence of cortistatin analyzed by western blotting, normalized to GAPDH levels and expressed relative to oxLDL-treated cells (dashed horizontal line). n = 7 independent experiments. *p < 0.05, **p < 0.001 *vs* oxLDL-treated cells. (**C**) Efflux of [^3^H]-cholesterol by macrophages exposed to oxLDL in the absence or presence of cortistatin. n = 3 independent experiments, in duplicates. (**D**) Gene expression of ABCA1 and PPARγ in aortas isolated from PBS-treated (control) or cortistatin-treated (CST) atherosclerotic apoE^−/−^ mice fed a high-fat diet during 16 weeks. n = 5 mice/group. (**E**) Gene expression of CD206, CD163, PPARγ and TNFα by peritoneal macrophages isolated from PBS-treated (control) or cortistatin-treated (CST) atherosclerotic apoE^−/−^ mice fed a high-fat diet during 16 weeks. n = 5 mice/group. (**F**) Gene expression of CD206 in aortic arch isolated from PBS-treated (control) or cortistatin-treated (CST) atherosclerotic apoE^−/−^ mice fed a high-fat diet during 16 weeks. n = 5 mice/group. *p < 0.05, **p < 0.001.

The accumulation of lipid-laden macrophage foam cells in the intima is an early event in the pathogenesis of atherosclerosis^1^,^2^,^3^. Infiltrating monocyte-derived macrophages ingest oxidized lipoproteins retained in subendothelial space of medium-large vessels. Lipoprotein uptake promotes the intracellular accumulation of various lipids, including cholesterol, in form of lipid droplets. This accumulation causes the macrophages to become foam cells and induces an inflammatory response. Therefore, we tested whether cortistatin regulated lipid accumulation in macrophages exposed to oxLDL. Cortistatin decreased the percentage of macrophages with mild and high load of lipid droplets (Fig. 6A). Cortistatin did not significantly affect the expression of CD36 and SRB1, two scavenger receptors involved in uptake of oxLDL by macrophages (Fig. 6B), arguing against this mechanism as one mainly involved in the effect of cortistatin on foam cell formation. However, cortistatin increased a 38% the efflux of cholesterol to extracellular apolipoprotein AI (ApoAI) in oxLDL-activated macrophages (Fig. 6C). Because this effect depended on the presence of ApoAI in the culture, the membrane transporter ATP biding cassette A1 (ABCA1) emerged as a potential mediator of the effect of cortistatin in cholesterol efflux^21^. Indeed, cortistatin increased the expression of ABCA1 in oxLDL-activated macrophages (Fig. 6B). In agreement, cortistatin also augmented the expression of peroxisome proliferator-activated receptor-γ (PPARγ), an inducer of ABCA1 expression^21^, in oxLDL-activated macrophages (Fig. 6B). Noteworthy, we found that the expression of ABCA1 and PPARγ relative to the macrophagic marker CD68 was higher in aortas isolated from cortistatin-treated apoE^−/−^ mice than in arteries isolated from untreated apoE^−/−^ mice (Fig. 6D), suggesting that the effect observed for cortistatin in foam cells *in vitro* could be also exerted *in vivo*. Moreover, peritoneal macrophages isolated from atherosclerotic apoE^−/−^ mice treated with cortistatin showed increased mRNA expression of PPARγ (Fig. 6E). Beside its role in ABCA1 induction, PPARγ is also involved in the expression of CD206, a marker of alternatively-activated M2 macrophages, which play a protective effect in atherosclerosis^22^,^23^,^24^,^25^. Thus, we found that treatment with cortistatin increased the expression of CD206, and other M2 markers such as CD163, in peritoneal macrophages of atherosclerotic apoE^−/−^ mice, at the same time that decreased the expression of TNFα, a marker of inflammatory macrophages (Fig. 6E). Interestingly, aortas isolated from cortistatin-treated apoE^−/−^ mice showed three-fold more expression of CD206 relative to CD68 than those isolated from untreated atherosclerotic mice (Fig. 6E), suggesting that this neuropeptide favoured the presence of M2 macrophages in developing atherosclerotic plaques.

## Discussion

Cortistatin is a neuropeptide that has recently emerged as a potent anti-inflammatory factor with capacity to regulate self-reactive responses in various experimental models of autoimmune disorders^14^,^16^,^17^,^18^. In this study, we provide evidence that cortistatin could be considered a protective therapy for cardiovascular disorders that course with exacerbated inflammatory and autoimmune responses, such as atherosclerosis. Using well-characterized mouse models of acute and chronic atherosclerosis, we demonstrate that cortistatin ameliorated plaque formation. Our data indicate that the effects of cortistatin are mainly exerted during the progression phase of the disease where innate and adaptive immune responses play pivotal roles. Whereas cortistatin did not significantly reduce the circulating levels of cholesterol in hyperlipidemic apoE^−/−^ mice, it regulates various pathologic components of atherosclerosis, including T cell-driven inflammation against arterial wall, the formation of foam cells and the binding of immune cells to endothelial cells. Future work must address whether cortistatin is able to reduce plaque size in animals with established atherosclerotic lesions and whether it is more effective in animals in which high-fat diet is interrupted.

Numerous studies in human and animals models indicate that Th1 cells and IFNγ enhance development of atherosclerotic lesions and contribute to lesion rupture through various biological effects. These include induction of adhesion molecule and chemokine expression by endothelial cells, stimulation of secretion of proinflammatory cytokines, chemokines, reactive oxygen species and matrix metalloproteinases by macrophages and inhibition of cholesterol efflux from foam cells^1^,^2^,^20^,^26^,^27^,^28^,^29^,^30^,^31^,^32^. On the other hand, like Th1 cells, Th17 cells are present in human atherosclerotic plaques^33^,^34^, and the involvement of Th17 cells in the progression of atherosclerosis has been largely demonstrated in experimental models, especially during early steps^35^,^36^, although some controversy exists^37^.Treatment with cortistatin diminished the presence of Th1 and Th17 cells and their derived cytokines in arteries and aortic sinus bearing atherosclerotic plaques. This effect could be mainly mediated at peripheral level in lymphoid organs, because treatment with cortistatin diminished the number of Th1 and Th17 cells in lymph nodes that drain atherosclerotic carotid artery, heart and aortic arch. However, as discussed below, cortistatin would be also impairing the entry of Th1 cells into the plaque by regulating the capacity of endothelial cells to bind T cells and macrophages. Even once within arterial wall, atherogenic T cells could be locally deactivated by cortistatin. Whether cortistatin regulates activation and/or expansion of atherogenic T cell clones acting directly on T cells or indirectly on antigen presenting cells is still unknown. Previous studies support an action of cortistatin on T cells and argues against any effect on dendritic cells^5^,^14^,^16^,^17^,^18^. Moreover, recent studies have associated the effect of cortistatin to the generation of Treg cells^14^,^18^. We also observed significant changes induced by cortistatin in this cell population in arteries and DLNs of atherosclerotic mice, at least at early stages of the disease, promoting a beneficial Treg/Th1 balance. It has been demonstrated that peripheral activation/generation of Treg cells and their recruitment to atherosclerotic plaque limits the progression of the lesion in experimental models^2^,^3^.

Beside T cells, accumulation of foam cells in the intima layer is critical in the progression of atheromatous plaque^1^,^2^,^3^. We observed that cortistatin impaired the formation of foam cells in response to the atherogenic factor oxLDL *in vitro*. This could be also occurring *in vivo*, because cortistatin-treated animals showed reduced lipid-loaded macrophages in the atherosclerotic lesion area. This effect is mediated by an increase in the efflux of cholesterol from macrophages, but not by a decrease in oxLDL uptake, because cortistatin did not significantly affect the expression of scavenger receptors involved in the uptake of cholesterol. Previous studies identified cortistatin as a potent regulator of macrophage function. Cortistatin regulates the secretion of a plethora of inflammatory mediators by activated macrophages, including inflammatory cytokines, chemokines and enzymes^8^,^16^,^17^,^18^. The present study is the first describing the action of cortistatin in the metabolism of cholesterol in macrophages. Although the precise molecular mechanisms involved in such effect remain unknown, our data indicate that cortistatin significantly increases the expression of the membrane transporter ABCA1 in macrophages, a protein critically involved in promoting cholesterol efflux from macrophages to extracellular acceptors^21^. Moreover, cortistatin up-regulates PPARγ, a transcription factor that up-regulates ABCA1 in macrophages^21^. Interestingly, both factors are increased in aortas isolated from cortistatin-treated atherosclerotic mice, supporting a link between these cholesterol efflux-inducing factors and the reduced numbers of aortic foam cells observed in animals treated with cortistatin. In agreement with these findings, it was previously reported that the stable agonist of ghrelin-receptor hexarelin stimulated the metabolic cascade involving PPARγ-ABCA1 in monocytes and macrophages and ameliorated the formation of atherosclerotic plaques in apoE^−/−^ mice^38^. Interestingly, many of the effects described for cortistatin in macrophages are mediated through its binding to ghrelin receptor^5^. Although further pharmacological studies are needed to identify specific receptors and signalling involved in the therapeutic action of cortistatin in atherosclerosis, previous reports suggested the capacity of cortistatin to bind to two different types of receptors, i.e., ghrelin-receptor and somatostatin-receptors, supporting the higher therapeutic effect observed for cortistatin in comparison to somatostatin or ghrelin in autoimmune and inflammatory disorders, vascular remodelling or pain^8^,^11^,^14^,^15^,^16^,^17^,^18^,^39^. Noteworthy, despite their effects were not compared in the same study, cortistatin shows higher efficiency than hexarelin in ameliorating chronic atherosclerosis^38^. On the other hand, the induction of PPARγ in macrophages by cortistatin could be also related with their conversion to a M2-like phenotype, as we observed a correlation between increased expression of PPARγ and M2 markers in peritoneum and aortas of cortistatin-treated apoE^−/−^ mice. This could have important therapeutic implications because various studies demonstrated the involvement of M2 macrophages in the inhibition atherosclerotic plaque progression^22^,^23^,^24^,^25^. In this sense, preliminary experiments from our laboratory support the capacity of cortistatin to favor the generation of M2 macrophages *in vitro* (unpublished data).

On the other hand, various *in vitro* and *in vivo* experiments of this study indirectly suggest that cortistatin could regulate the entry of inflammatory monocytes and lymphocytes to the plaque by impairing their binding to arterial endothelium. This effect could be mediated by decreasing the expression of adhesion molecules, mainly P-selectin and E-selectin, in endothelial cells activated in an atherogenic environment. Evidence demonstrated that P-selectin and E-selectin together play an important role in both early and advanced stages of atherosclerotic lesion development^40^,^41^. Moreover, macrophages and Th1 cells treated with cortistatin did not significantly change their capacity to bind to endothelial cells and to migrate to chemotactic stimuli, supporting that the effect of cortistatin is exerted at the endothelial level. Various studies indicate that endothelial cells express receptors for cortistatin and that they are able to respond to this neuropeptide^42^,^43^.

Finally, we recently reported that cortistatin limits proliferation and migration of SMCs in response to platelet-derived growth factor, which is produced by endothelial cells, macrophages and SMCs in the atherosclerotic plaque^11^. Moreover, cortistatin diminished the formation of neointimal lesions and prevented arterial stenosis in non-atherogenic conditions, but subjected to blood flow alteration and vascular injury^11^. The present study shows that treatment with cortistatin regulated the presence of SMCs in the plaque and intimal/medial ratio in carotid and aorta, suggesting that cortistatin could limit the outward vascular remodelling observed during the progression of atherosclerosis. However, it remains to determine whether this effect on SMCs could affect the stability of the mature plaque in advanced atherosclerosis.

An important question that needs to be addressed is which role is played by endogenous cortistatin in the regulation of atherosclerosis. Indirect evidence indicates that cortistatin must exert a protective role. Cortistatin is produced by arterial endothelium and SMCs and its expression increased in activated SMC cultures and in neointimal SMCs of arteries subjected to blood-flow alterations^11^. Moreover, cortistatin is expressed by human atheromas, specifically localized in SMCs and infiltrating macrophages^11^. Accordingly with its beneficial effect in vascular and immune disorders, animals lacking cortistatin responded with exacerbated neointima formation upon carotid ligation^11^, and macrophages and T cells isolated from cortistatin-deficient mice showed increased inflammatory and self-reactive responses^18^, supporting a role of cortistatin as an endogenous break of immune and vascular responses. Therefore, cortistatin-deficient mice should be more susceptible to suffer inflammatory vascular disorders. However, these animals showed an unexpected phenotype when subjected to various inflammatory and autoimmune disorders, such as sepsis, autoimmune encephalomyelitis or colitis, showing less incidence and severity than wild-type mice^18^. This paradoxical effect is due to overproduction of glucocorticoid by a superactivated hypothalamus-pituitary-adrenal axis^18^, which makes complex evaluating the endogenous role of cortistatin in inflammatory cardiovascular disease.

In summary, we describe for the first time that, despite the unstability of cortistatin in body fluids (see Methods), this peptide could be highly efficient in the treatment of inflammatory cardiovascular disorders. Its multimodal action on various components of the disease would suppose a therapeutic advantage versus current treatments. Because the effects observed are based on mouse models of atherosclerosis, extrapolations to clinical practice have to be made with caution. Notably, injection of cortistatin has been proven safe (no side effects were observed) and effective in the treatment of patients with Cushing’s disease^44^. These findings encourage further studies aimed to assess whether cortistatin can be used as a pharmaceutical agent to prevent the clinical manifestations of atherosclerosis in combination with therapies to lower plasma cholesterol.

## Methods

### Ethic statement

The experiments reported in this study followed the ethical guidelines for investigations of experimental animals approved by the Ethical Committee of Spanish Council of Scientific Research and performed in accordance with the guidelines from Directive 2010/63/EU of the European Parliament on the protection of animals used for scientific purposes.

### Experimental model of acute atherosclerosis

To induce acute atherosclerosis in hyperlipidemic mice, we subjected female C57Bl/6 apoE^−/−^ mice (6 weeks-old, Charles River) to partial ligation of left carotid artery. Anesthesia was induced by intraperitoneal injection of xylazine (10 mg/kg) and ketamine (80 mg/kg) mixture. A ventral midline incision (5 mm) was made in the neck and three of four caudal branches of left carotid artery (external carotid, internal carotid and occipital artery) were ligated with 6–0 silk suture, leaving the superior thyroid artery intact (Supplementary Fig. S1A). One day after surgery, animals were fed a high-fat Western diet (38% fat, 0.15% cholesterol, diet U8958, SAFE) and received intraperitoneally PBS (control) or mouse cortistatin-14 (1 nmol, which corresponds to 1.7 μg of cortistatin-14, obtained from American Peptides) every two days for a period of three weeks. The dose of cortistatin used in this study was selected in base of results obtained in previous experiments performed in other autoimmune and vascular models^11^,^14^,^15^,^16^,^17^,^18^. Serum levels of cortistatin elevated from 32 pg/ml (basal levels before injection) to 62 and 55 pg/ml at 10 min and 30 min after its intraperitoneal injection, respectively, and back to basal levels 1 hour later. When indicated, naïve female apoE^−/−^ mice (9 weeks-old) fed a normal chow diet were used as reference. Mice were sacrificed with carbon dioxide 14 or 21 days after surgery. Some animals were subsequently perfused with cold-PBS and with 4% paraformaldehyde/0.1 M phosphate buffer pH 7.4 (buffered-PFA), and the ligated and contralateral unligated arteries were isolated for morphometry and immunofluorescence analysis. In other animals, ligated and unligated carotids were isolated for protein and gene expression determination, inflammatory infiltration analysis and *ex vivo* artery culture. Moreover, we isolated the ipsilateral and contralateral deep cervical lymph nodes (used as DLNs of carotid artery)^45^,^46^,^47^,^48^, spleen and mesenteric lymph nodes (used as non-DLN controls) for flow cytometric analysis. For morphometric and immunofluorescence analysis, we fixed the isolated carotid arteries in 4% buffered-PFA for 6 hours, and then subjected them to cryopreservation in 30% sucrose/0.1 M phosphate buffer at 4 °C, embedding in OCT-compound and freezing. We obtained a series of 20 cross cryosections (6 μm thick) every 50 μm over a 1 mm length of carotid artery beginning at 0.5 mm from carotid bifurcation. We measured the area occupied by atherosclerotic lesion and by arterial medial layer in five carotid sections stained with hematoxylin/eosin (corresponding to divisions 1, 5, 9, 13 and 17 of the series) and in five carotid sections stained with Oil Red O (corresponding to divisions 3, 7, 11, 15 and 19 of the series) in a blinded manner using a Zeiss microscope and the ImageJ software. The mean for each of these 10 divisions (5 hematoxilyn/eosin- and 5 Oil-Red O-stained sections) was calculated for both arteries from every animal. Volume of the plaque on 1 mm length of carotid was calculated by the sum of plaque areas in each of these 10 divisions multiplied by 50 (50 is the length in μm between divisions)^49^. Moreover, we analyzed the infiltration of inflammatory cells in the plaques in the other collected 10 carotid cryosections by immunostaining with fluorescence-labelled antibodies against CD4, CD68 and αSMA as described below. Furthermore, to determine the number and phenotype of infiltrating cells in ligated and unligated carotid (isolated at day 21 post-ligation), a single-cell suspension was obtained by digestion of artery in a mixture of collagenases I and XI, DNase I and hyaluronidase (1 hour, 37 °C) as previously described^50^,^51^ and then analyzed by flow cytometry as described below.

To determine the production of cytokines, segments of unligated and ligated carotid arteries isolated from untreated and cortistatin-treated atherosclerotic mice at day 21 were *ex vivo* cultured in complete DMEM (DMEM supplemented with 100 U/ml penicillin/streptomycin, 2 mM L-glutamine and 10% fetal calf serum) in the presence of phorbol 12-myristate 13-acetate (PMA, 25 ng/ml) plus ionomycin (0.5 μg/ml) in 24-well plates at 37 °C and 5% CO_2_. When indicated, cortistatin-14 (100 nM) was added to PMA/ionomycin-activated cultures of ligated carotid arteries isolated from untreated atherosclerotic mice at day 21. After 24 hours, we determined the cytokine contents in culture supernatants by using specific sandwich ELISAs (BD Pharmingen). Moreover, total proteins were obtained from ligated and unligated carotid arteries isolated from untreated and cortistatin-treated atherosclerotic mice at day 21 as previously described^18^, and the content of cytokines in protein extracts was determined by using specific sandwich ELISAs (BD Pharmingen).

### Experimental model of chronic atherosclerosis

To induce chronic atherosclerosis, we fed female apoE^−/−^ mice (6 weeks-old) with a high-fat Western diet and water *ad libitum* for 16 weeks. Animals received intraperitoneally PBS (control) or mouse cortistatin-14 (1 nmol) three times per week during 15 weeks starting one week after initiation of Western diet. We monitored plasma cholesterol (Accuntred Plus kit, Roche) and glucose levels (One Touch Verio, LifeScan) every four weeks in blood samples collected from the tail, and measured body weight and food consumption every week. When indicated, naïve female C57Bl/6 mice (22 weeks-old) fed a normal chow diet were used as reference. Ten or 16 weeks after initiating a Western diet, mice were sacrificed with carbon dioxide and spleen, lymph nodes draining heart, aortic arch and aorta (deep cervical, cranial mediastinal, lumbar aortic and medial iliac lymph nodes)^48^ and mesenteric lymph nodes (used as non-DLN controls) were collected for flow cytometric analysis. In some animals, artery segments containing aortic sinus and aortic arch were dissected at week 16, and processed for determination of RNA expression or digested^45^,^46^ for flow cytometric characterization of inflammatory cell infiltration as described below. Moreover, the descending aorta comprising from proximal ascending thoracic aorta to iliac bifurcation was microdissected *in situ* at week 16 and the atherosclerotic plaques were quantified after Sudan IV staining as described below. Some animals were sacrificed at week 16, perfused with cold-PBS and 4% buffered-PFA, and the segments containing heart-aortic sinus and aortic arch were isolated, fixed in 4% buffered-PFA for 6 hours, cryopreserved in sucrose, embedded in OCT-compound, cryosectioned and processed for morphometry and immunofluorescence analysis (see below). Furthermore, mice were anesthetized with isofluorane at weeks 10 and 16, and the peritoneal lavage was collected in cold-DMEM. Macrophages were isolated by plastic adherence of the peritoneal suspension by incubation during 3 hours at 37 °C in complete DMEM and then used to quantify gene expression and for assaying cell adhesion and migration as described below.

### Sudan IV and Oil Red O staining

Descending aorta was fixed in 4% buffered-PFA, depleted of adventitial fat and stained with 5% Sudan IV (dissolved in 50% acetone/35% ethanol) for 15 minutes, destained with 80% ethanol for 5 minutes, extensively washed in tap water during 1 hour, pinned flat onto a white rubber board, opened longitudinally using a dissecting microscope and photographed. Total aortic area and Sudan IV-positive lesion area were quantified in the images using ImageJ software.

Transversal and longitudinal cryosections (8 μm thick) of aortic sinus and aortic arch were sequentially obtained and processed for hematoxylin/eosin staining or for Oil Red O staining. For Oil Red O staining, sections were rinsed with 60% isopropanol for 5 minutes, stained with 0.5% Oil Red O/60% isopropanol (20 °C, 10 minutes), destained with 60% isopropanol for 2 minutes and extensively washed with distiller water. Finally, nuclei were counterstained with hematoxylin. Images of stained sections were acquired in a Zeiss microscope and the area occupied by the plaques in aortic arch and sinus was measured in a blinded fashion using the ImageJ software.

### Flow cytometric analysis

For analysis of artery-infiltrating cells, single-cell suspensions were isolated 21 days after carotid ligation or 16 weeks after initiating a high-fat diet (see above) and were incubated with anti-2.4 G2 antibody (Mouse BD Fc Block™, 1:100, 4 °C, 10 minutes) to avoid non-specific binding to Fc-receptors and with 7-Aminoactinomycin D (Calbiochem, 1:100) to exclude dead cells. After washing in PBS/0.1% BSA, cells were surface stained with allophycocyanin-labelled anti-CD4, FITC-labelled anti-CD11b and phycoerythrin-labelled anti-CD45 monoclonal antibodies (each at 4–5 μg/ml, BD Bioscience, 30 minutes, 4 °C) and were analyzed in a FACScalibur flow cytometer (BD Biosciences). Data were acquired until at least 100.000 events were collected from a live gate using forward/side scatter plots and 7-Aminoactinomycin D staining.

To determine the number of cells expressing cytokines, infiltrating inflammatory cells isolated from ligated and unligated carotids at day 21 after ligation and from aortic sinus and aortic arch from apoE^−/−^ mice at week 16 after initiating a high-fat diet, as well as spleen, DLN and non-DLN cells isolated from apoE^−/−^ mice at days 10 and 21 after carotid ligation or at week 16 after initiating a high-fat diet were activated with PMA (25 ng/ml) for 14 hours in the presence of monensin (1.33 μM) for the last 6 hours. Cells were then incubated with anti-2.4 G2 antibody plus 7-Aminoactinomycin D, washed in PBS/0.1% BSA and stained with allophycocyanin-labelled anti-CD4 monoclonal antibody as described above. After extensive washing, cells were fixed/permeabilized with Cytofix/Cytoperm solutions (BD Biosciences), stained with phycoerythrin-labelled anti-IL-17 and FITC-labelled anti-IFNγ monoclonal antibodies (BD Pharmingen, 2 μg/ml, 30 minutes, 4 °C) and analyzed in a FACScalibur flow cytometer.

For FoxP3 staining, carotid cell isolates and DLN cells were isolated from mice 14 and 21 days after carotid ligation or 10 and 16 weeks after initiating a high-lipid diet and incubated with FITC-labelled anti-CD25 and allophycocyanin-labelled anti-CD4 monoclonal antibodies (5 μg/ml, BD Biosciences) for 1 hour at 4 °C. After extensive washing, cells were fixed/permeabilized (eBioscience), stained with phycoerythrin-labeled anti-FoxP3 antibody (5 μg/ml, eBioscience) for 30 minutes at 4 °C and analyzed in a FACScalibur flow cytometer. In all cases, we used isotype-matched antibodies (BD Biosciences) as controls.

### Determination of lipid accumulation and cholesterol efflux in macrophages

Macrophages were generated by differentiating bone marrow cell precursors isolated from untreated C57Bl/6 mice (sacrificed by carbon dioxide) and then incubated in complete DMEM in the presence of macrophage-colony stimulating factor (Preprotec, 20 ng/ml) for 6–8 days. To determine the accumulation of lipid droplets, macrophages (5 × 10^5^) were seeded in coverslips (8 hours, 37 °C, in complete DMEM) and incubated in DMEM supplemented with 100 U/ml penicillin/streptomycin, 2 mM L-glutamine and 2% fetal calf serum in the presence of oxLDL (50 μg/ml, KALEN Biomedical) and mouse cortistatin-14 (100 nM). After 24 hours, cells were fixed in 4% buffered-PFA (10 minutes, 22 °C) and intracellular neutral lipids were stained with Oil Red O (0.15%, 15 minutes, 22 °C). After extensive washing in water, macrophages were counterstained with hematoxylin and observed in a Zeiss microscope. Images were analyzed with the ImageJ software to quantify the content of intracellular lipid droplets per cell and determine the percentage of foam cells.

To determine the cholesterol efflux, macrophages (10^5^) were cultured in 96-well plates with complete DMEM. After 16 hours, cells were incubated in DMEM-BSA (DMEM supplemented with 100 U/ml penicillin/streptomycin, 2 mM L-glutamine and 0.2% fatty acid-free bovine serum albumin) in the presence of oxLDL (50 μg/ml), [^3^H]-cholesterol (0.5 μCi/ml, PerkinElmer) with or without mouse cortistatin-14 (100 nM) for 10 hours. Cells were then washed with DMEM-BSA and incubated again with ApoAI (10 μg/ml, Sigma) with or without cortistatin (100 nM). After 10 hours, culture supernatants were collected and cells lysed in NaOH (0.1 M, 5 hours, 22 °C). The cpm in cell lysates (lys) and supernatants (sup) were quantified in a MicroBeta Trilux counter, and the ApoAI-mediated efflux of cholesterol was determined by using the following formula: % efflux = (cpm sup/cpm sup + lys) × 100.

### Immunofluorescence analysis

Sections of carotids, aortic sinus and aortic arch were fixed in 4% buffered-PFA (10 minutes, 20 °C), blocked with 10% goat serum/0.2% Triton X-100/1% BSA (45 minutes, 20 °C) and incubated with rabbit anti-mouse CD68 antibody (BD Bioscience, dilution 1:500) or rat anti-mouse CD4 antibody (BD Bioscience, dilution 1:1000) and mouse anti-mouse αSMA antibody (Sigma, dilution 1:1000) overnight at 4 °C. After extensive washing with PBS/1% BSA, sections were incubated with Alexa Fluor 546-conjugated goat anti-rabbit antibody, Alexa Fluor 488-conjugated goat anti-rat antibody, Alexa Fluor 488-conjugated goat anti-mouse antibody, or Alexa Fluor 594-conjugated goat anti-mouse antibody (Life Biotechnology, 1 hour, 20 °C, all diluted at 1:1000 in PBS/2% goat serum/0.1% Triton X-100). Nuclei were Hoechst-counterstained and slices examined in an Olympus fluorescence microscope. Sections in which we omitted primary antibodies were used as negative controls. We quantified the area occupied by florescence-stained cells in the plaque of arteries and aortic sinus using the ImageJ software.

### Determination of gene expression by real-time PCR

Total RNA was isolated from carotid arteries, aortas and peritoneal macrophages following the manufacturer’s protocol (Tripure, Roche). Precipitated RNA was treated with DNase 1 (Sigma-Aldrich) before reverse transcription (RevertAid First Strand cDNA Synthesis Kit, Thermo Fisher Scientific). SYBER green quantitative PCR (SensiFast Sybr No-Rox mix, Bioline) was performed on the Bio-Rad CFX using the following conditions: 95 °C for 5 minutes followed by 35 cycles at 95 °C for 30 seconds, annealing (see Supplementary Table S1 for annealing temperature and time used for each gene) and extension at 72 °C for 30 seconds. Primer sequences are listed in Supplementary Table S1. The expression of each gene was normalized against the expression of the housekeeping gene GAPDH in every PCR reaction.

### Western blot analysis

Mouse macrophages differentiated from bone marrow were cultured in complete DMEM in the absence or presence of oxLDL (50 μg/ml) and mouse cortistatin-14 (100 nM). After 16 hours, cells were lysed by incubation with lysis buffer (50 mM Tris-HCl pH 7.5, 150 mM NaCl, 1 mM EDTA, 1% Triton X-100, 1% sodium deoxycholic acid, 0.1% SDS and 10 μg/ml of a cocktail of proteinase inhibitors) for 30 minutes on ice. Proteins extracts (20 μg/lane) were separated on 7.5% SDS-polyacrylamide gels and blotted onto polyvinylidene difluoride membranes (Millipore) using a semidry system. Membranes were blocked with TBS-Tween-20/5% non-fat dry milk for 1 hour at room temperature and subsequently probed overnight at 4 °C with primary rabbit anti-mouse CD36 or ABCA-1 antibodies (at 1/1000; Novus Biologicals), rabbit anti-mouse PPARγ or SRB1 antibodies (at 1/500, Cell Signaling) or rabbit anti-mouse GAPDH antibody (at 1/5000, R&D System). Immunodetection was performed by incubation with a peroxidase-conjugated anti-rabbit antibody (at 1:5000, 2 hours, 20 °C, Dako-Cytomation) and developed with enhanced chemiluminescence detection system (ECL plus, Amersham). Protein expression was represented as % of expression in densitometry units relative to GAPDH.

### Adhesion assay of macrophages, lymphocytes and Th1 cells to whole aorta

The macrophage and lymphocyte adhesion assay was performed as previously described^35^. Aortas from untreated and cortistatin-treated ApoE^−/−^ mice fed a high-fat diet for 10 weeks were dissected out, opened longitudinally and pinned to a sterile agarose gel in serum-free DMEM. In parallel, peritoneal macrophages and DLN lymphocytes were isolated from ApoE^−/−^ mice after 16 weeks with Western diet, and macrophages were differentiated from bone marrow cell precursors of C57Bl/6 mice (see above). Peritoneal macrophages, bone marrow-derived macrophages and DLN lymphocytes were labelled with calcein-AM (10 μM, Molecular Probes) following manufacturer’s recommendations. Calcein-labelled cells (10^6^ cells) were co-incubated with pinned aortas in complete DMEM for 1 hour at 37 °C. Nonadhered cells were washed off with PBS and the number of cells firmly adhered to the aorta were counted in three consistent fields using a fluorescent microscope (Zeiss AxioObserver Z1). The areas counted were identified within a 100 mm^2^ grid, and the same locations per aorta were counted by a blinded observer. In some experiments, aortas isolated from apoE^−/−^ mice fed a high-fat diet for 10 weeks were pretreated with cortistatin (100 nM) for 12 hours in complete DMEM at 37 °C before the addition of macrophages. Alternatively, macrophages were preincubated with cortistatin (100 nM) in complete DMEM for 6 hours before their addition to pinned aortas.

Furthermore, CD4^+^ cells (10^6^/ml) isolated from spleen of C57Bl/6 mice (sacrificed by carbon dioxide) by immunomagnetic negative selection (Invitrogen) were differentiated to Th1 cells (purity > 94% IFNγ^+^ cells) by stimulation during 5 days with anti-CD3/anti-CD28 antibodies (5 μg/ml, BD Pharmingen) in the presence of IL-2 (25 U/ml, PreproTech), IL-12 (20 ng/ml, PreproTech) and anti-IL4 antibody (5 μg/ml, BD Pharmingen), as previously described^52^. Cortistatin (100 nM) was added to Th1 cells at days 0 and 3 of culture. Th1 cells differentiated in the absence or presence of cortistatin were Hoechst-stained and added to aorta arteries from C57Bl/6 mice (8 weeks-old) that were previously opened longitudinally, pinned in sterile agar and activated during 6 hours with IL-1β (20 ng/ml) and TNFα (100 ng/ml) in complete RPMI medium. After 45 minutes of incubation at 37 °C and 5% CO_2_, aortas were extensively washed with PBS and scanned by spinning disk microscopy (Olympus). The total number of Th1 cells bound to aorta was determined by analysis of consecutive deconvolved images of the whole aorta using Methamorph software.

### Binding assay to adhesion molecule chimeras

Th1 cells differentiated in the absence or presence of cortistatin (see above) were diluted at 5 × 10^5^ cells/ml in PBS containing 0.1% BSA and 20 mM HEPES pH 7.4 and perfused over P-selectin or E-selectin chimera-coated coverslips (5 μg/ml, R&D Systems) in parallel plate flow chambers (Harvard Apparatus). We applied a laminar flow rate of 1 dynes/cm^2^ (a share that corresponds to aortic arch). We recorded during one minute the number of Th1 cell interactions with selectin chimeras with a phase contrast objective (20x)and a microscope connected to Videolab software (Ed Marcus Laboratories).

### Determination of binding of monocytes to endothelial cells

Human umbilical vein endothelial cells (HUVECs, Clonetics) were cultured (10^5^ cells/well, in 48-well plates) in complete EGM-2 medium (Clonetics) containing 20% FBS in the presence of oxLDL (50 μg/ml) and/or various concentrations of human cortistatin-14 for 24 hours. Moreover, HUVEC monolayers were extensively washed with EGM-2 medium and co-incubated with 10^4^ calcein AM-labelled THP1 human monocytic cells (ATCC) for 1 hour. After extensive washing, the number of THP1 cells bound to HUVEC monolayer was determined in a fluorescence microscope.

### Determination of macrophage migration

Macrophage migration was measured using a modified Boyden’s chamber method. Macrophages from untreated or cortistatin-treated ApoE^−/−^ mice were isolated from peritoneal lavage after 16 weeks on a high-fat diet, suspended in complete DMEM medium and plated on Matrigel-coated 8 μm cell culture inserts (Millipore) at 5 × 10^4^ cells per insert. MCP-1 was added to a final concentration of 50 ng/ml to the lower chamber and the cells were incubated for 8 hours at 37 °C. Alternatively, macrophages isolated from untreated ApoE^−/−^ mice were isolated from peritoneal lavage after 16 weeks on a high-lipid diet and incubated with DMEM medium in the absence or presence of cortistatin (100 nM) in the upper insert 20 minutes before the addition of MCP-1 in the lower chamber. The inserts were then lifted, non-migrated cells on the upper surface of the membrane were removed with a cotton swab and the membrane was then fixed in methanol and stained with DAPI. The DAPI-positive cells on the lower surface of the membrane were counted under an inverted fluorescence microscope (Olympus) and the cell migration was expressed as the number of cells per field of view.

### Statistical analysis

All data are mean ± s.e.m. We analyzed data for statistical differences using Student’s t-test or one-way ANOVA followed by a test for multiple comparisons or with non-parametric Mann–Whitney U test for comparisons between two groups. We considered P-values < 0.05 (two-tailed) as significant.

## Additional Information

**How to cite this article**: Delgado-Maroto, V. *et al*. Cortistatin reduces atherosclerosis in hyperlipidemic ApoE-deficient mice and the formation of foam cells. *Sci. Rep.* **7**, 46444; doi: 10.1038/srep46444 (2017).

**Publisher's note:** Springer Nature remains neutral with regard to jurisdictional claims in published maps and institutional affiliations.

## Supplementary Material

Supplementary Tables and Figures
