# Supplementary material for: Cortistatin reduces atherosclerosis in hyperlipidemic ApoE-deficient mice and the formation of foam cells
Source: Sci Rep. 2017 Apr 13;7:46444. doi: 10.1038/srep46444 (PMC5390288; doi:10.1038/srep46444)
Supplement: Supplementary Tables and Figures [file srep46444-s1.pdf]

SUPPLEMENTARY INFORMATION

Cortistatin reduces atherosclerosis in hyperlipidemic ApoE-deficient mice and the formation of foam cells

Virginia Delgado-Maroto [1]; Raquel Benitez[1], Irene Forte-Lago[1], Maria Morell[1]; Elena Maganto-Garcia[2]; Luciana Souza-Moreira[1]; Francisco O'Valle[3]; Mario Duran-Prado[1,4]; Andrew H. Lichtman[2]; Elena Gonzalez-Rey[1*] and Mario Delgado[1*]

Supplementary Table 1. Sequences of primers and their temperature and time of annealing used for quantitative real-time PCR analysis (m, mouse; h, human).

Name	Sequence 5´---3´	Annealing Temperature Time
mTNFα-FW<br>mTNFα-RV	GCGACGTGGAACTGGCAGAAGAG<br>TGAGAGGGAGGCCATTTGGGAAC	64ºC<br>30 sec
mIFNγ-FW<br>mIFNγ-RV	ACACTGCATCTTGGCTTTGC<br>TTGCTGATGGCCTGATTGTC	58ºC<br>30 sec
mCD68-FW<br>mCD68-RV	CCATCCTTCACGATGACACCT<br>GGCAGGGTTATGAGTGACAGTT	60ºC<br>30 sec
mF4/80-FW<br>mF4/80-RV	CCCCAGTGTCCTTACAGAGTG<br>GTGCCCAGAGTGGATGTCT	60ºC<br>45 sec
mCD206-FW<br>mCD206-RV	CCAAATGATGAGCTGTGGATTG<br>GCTCTCCAGGAAGCCATTTA	60ºC<br>30 sec
mIL17-FW<br>mIL17-RV	CTGTGTCTCTGATGCTGT TG<br>ATGTGGTGGTCCAGCTTTC	60ºC<br>30 sec
mCD163-FW<br>mCD163-RV	TGACGACAACTTCAGCAAAGA<br>CCAGAACCAGCTCCCAATTTA	60ºC<br>30 sec
mPPARγ-FW<br>mPPARγ-RV	GCCCTTTGGTGACTTTATGGA<br>GCAGCAGGTTGTGTTGGATG	60ºC<br>30 sec
mABCA1-FW<br>mABCA1-RV	GGACTTGCCTTGTTCCGAGAG<br>GCTGCCACATAACTGATAGCGA	64ºC<br>30 sec
mICAM1-FW<br>mICAM1-RV	GTGATGCTCAGGTATCCATCCA<br>CACAGTTCTCAAAGCACAGCG	62ºC<br>30 sec
mGAPDH-FW<br>mGAPDH-RV	AACTTTGGCATTGTGGAAGG<br>ACACATTGGGGGTAGGAACA	according with each gene
mE-selectin-FW<br>mE-selectin-RV	ATGAAGCCAGTGCATACTGTC<br>CGGTGAATGTTTCAGATTGGAGT	62ºC<br>30 sec
mP-selectin-FW<br>mP-selectin-RV	CATCTGGTTCAGTGCTTTGATCT<br>ACCCGTGAGTTATTCCATGAGT	62ºC<br>30 sec
hP-selectin-FW<br>hP-selectin-RV	TGCATTAGTTGGACCGGAAG<br>AAAAGCAGTGAGCGGATGAA	61ºC<br>45 sec
hE-selectin-FW<br>hE-selectin-RV	TGGGTAGGAACCCAGAAACC<br>TCTCCACGCAGTCCTCATCT	61ºC<br>45 sec
hICAM1-FW<br>hICAM1-RV	AGCCAAGAGGAAGGAGCAAG<br>GCATACCCAATAGGCAGCAA	61ºC<br>45 sec

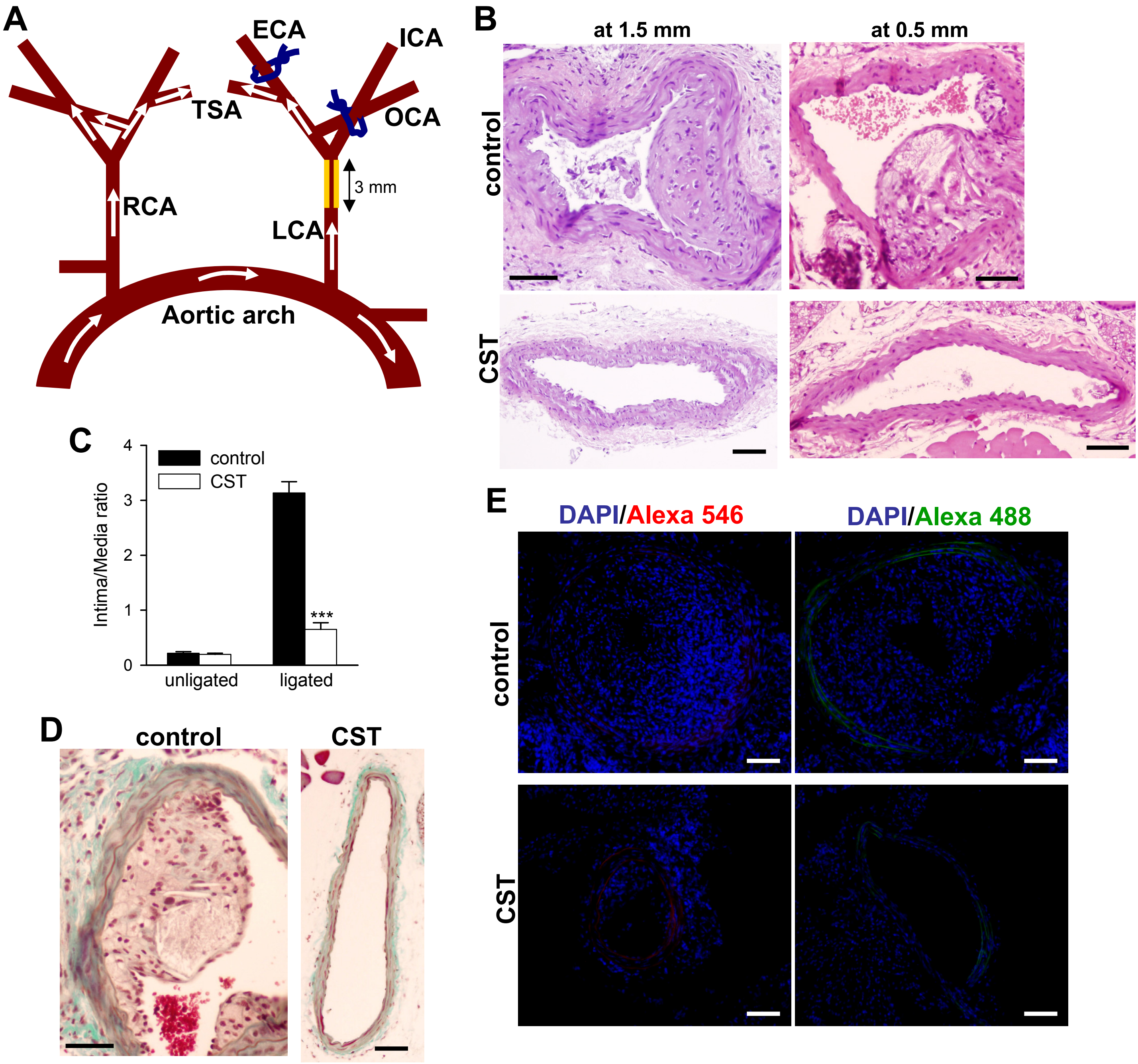

Supplementary Figure S1.- Cortistatin protects from acute atherosclerosis in carotid artery in hyperlipidemic mice. ApoE$^{-/-}$ mice subjected to partial ligation of left carotid artery and fed a high-fat diet were treated with PBS (control) or with cortistatin (CST) every other day starting one day after ligation. Contralateral unligated carotid and ligated carotid were collected two and three weeks after ligation. **A.** Scheme depicting the surgical procedure of partial ligation of left carotid artery. Partial ligation of left common carotid artery (LCA) was performed with a single 6-0 silk suture (A) near to the bifurcation in internal carotid artery (ICA) and external carotid artery (ECA). Partial ligation of LCA was performed by ligating with 6-0 silk suture three branches of the LCA (the external carotid artery ECA, the internal carotid artery ICA and the occipital artery OCA), while leaving the superior thyroid artery (STA) open. The contralateral right common carotid artery (RCA) was left unligated and served as control. Morphometric analysis was performed along the 3-mm section of LCA depicted by yellow lines. **B.** Carotid sections (at 0.5 or 1.5 mm from carotid bifurcation) were isolated 2 weeks after ligation and stained with hematoxylin-eosin. Scale bars: 100 μm. **C.** Intimal lesions and media layers were measured by morphometry in sections stained with hematoxylin-eosin and expressed as intima/media ratio values. n=14 mice per group, performed in three experiments. ***p<0.0001 *vs* control. **D.** Masson's trichromic staining of ligated carotid sections isolated 2 weeks after ligation revealing collagen deposition in media layer. Scale bars: 100 μm. **E.** Negative controls for immunofluorescence analysis in which primary antibodies were excluded and only secondary Alexa Fluor-labelled antibodies were used. Nuclei were Hoechst-counterstained. Scale bars: 100 μm.

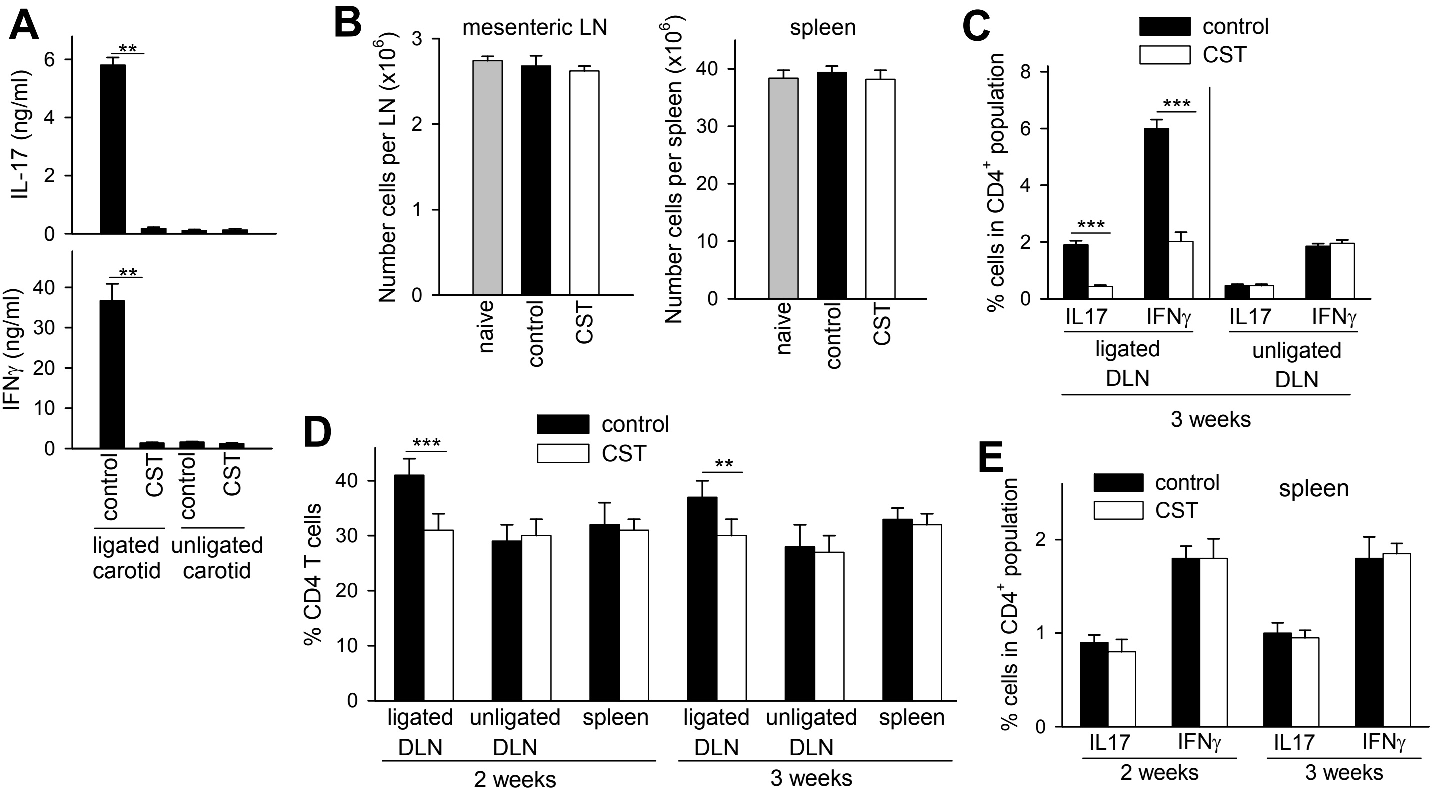

Supplementary Figure S2.- Cortistatin downregulates local and peripheral inflammatory and T-cell responses in acute atherosclerosis. ApoE$^{-/-}$ mice subjected to partial ligation of left carotid artery and fed a high-fat diet were treated with PBS (control) or with cortistatin (CST) every two days, starting one day after ligation. **A.** *Ex vivo* cytokine production by unligated and ligated carotids isolated 3 weeks after ligation. n=6 mice per group, performed in two independent experiments. **p<0.0001 *vs* control. **B.** Total cell numbers determined in mesenteric lymph nodes (used as non-DLN controls) and spleens. Naïve apoE$^{-/-}$ mice fed a normal chow diet were used as reference. n=5 mice per group. **C.** Flow cytometric analysis of IFNγ-producing CD4 T cells and IL-17-producing CD4 T cells in lymph nodes that drain (DLNs) right (unligated) and left (ligated) carotids collected 3 weeks after carotid ligation. n=6 mice per group, performed in two independent experiments. **D.** Flow cytometric analysis of CD4$^+$ lymphocytes in DLNs of unligated and ligated carotids and in spleens isolated 2 or 3 weeks after carotid ligation. n=6 mice per group. **E.** Percentage of IFNγ- and IL-17-producing cells in the CD4 population in spleens isolated 2 or 3 weeks after carotid ligation. **p<0.001; ***p<0.0001 *vs* control.

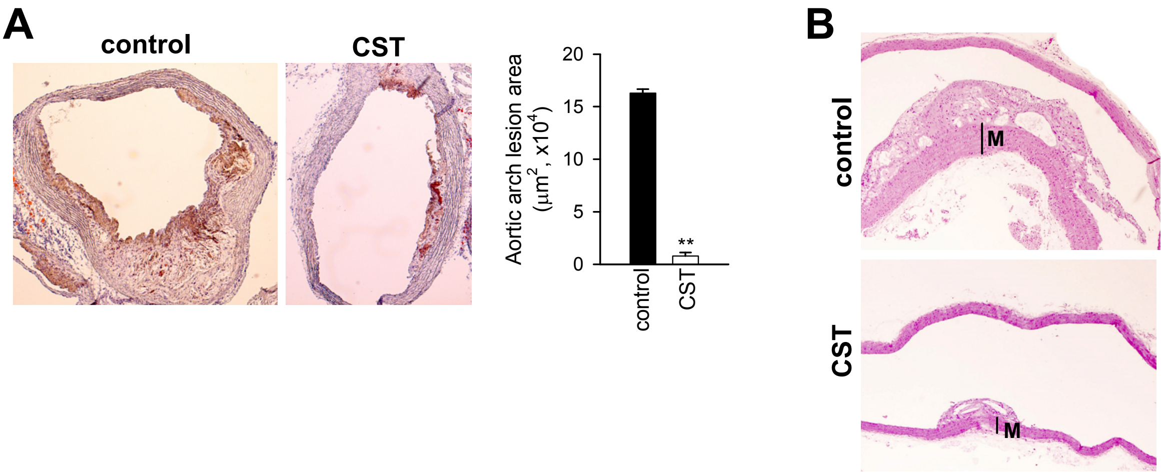

Supplementary Figure S3.- Effect of cortistatin in chronic atherosclerosis. ApoE$^{-/-}$ mice fed a high-fat diet during 16 weeks were treated with PBS (control) or cortistatin (CST) three times per week starting one week after initiation of diet. **A.** Images and area quantification of atherosclerotic plaques in aortic arch cross-sections stained with Oil Red-O. n=18 mice/group. **p<0.001 vs control. **B.** Images of sections of aortic arch stained with hematoxylin-eosin showing a decrease in medial muscle layer (marked with M) thickening in arteries isolated from cortistatin-treated mice compared to those isolated from untreated atherosclerotic mice.

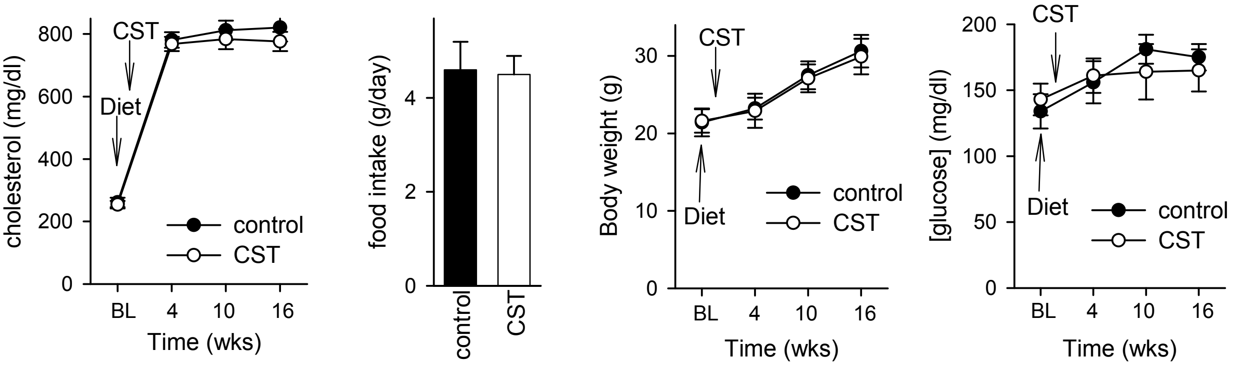

Supplementary Figure S4.- Effect of cortistatin in serum cholesterol and glucose levels and in body weight and food intake. ApoE$^{-/-}$ mice fed a high-fat diet during 16 weeks were treated with PBS (control) or cortistatin (CST) three times per week starting one week after initiation of diet. Levels of cholesterol (32 mice/group) and glucose (12 mice/group) were determined in blood collected from tail vein at different time points. Mean of daily food intake was determined for the entire period (16 weeks, n=18 mice/group) and body weight (n=18 mice/group) was determined at different time points.

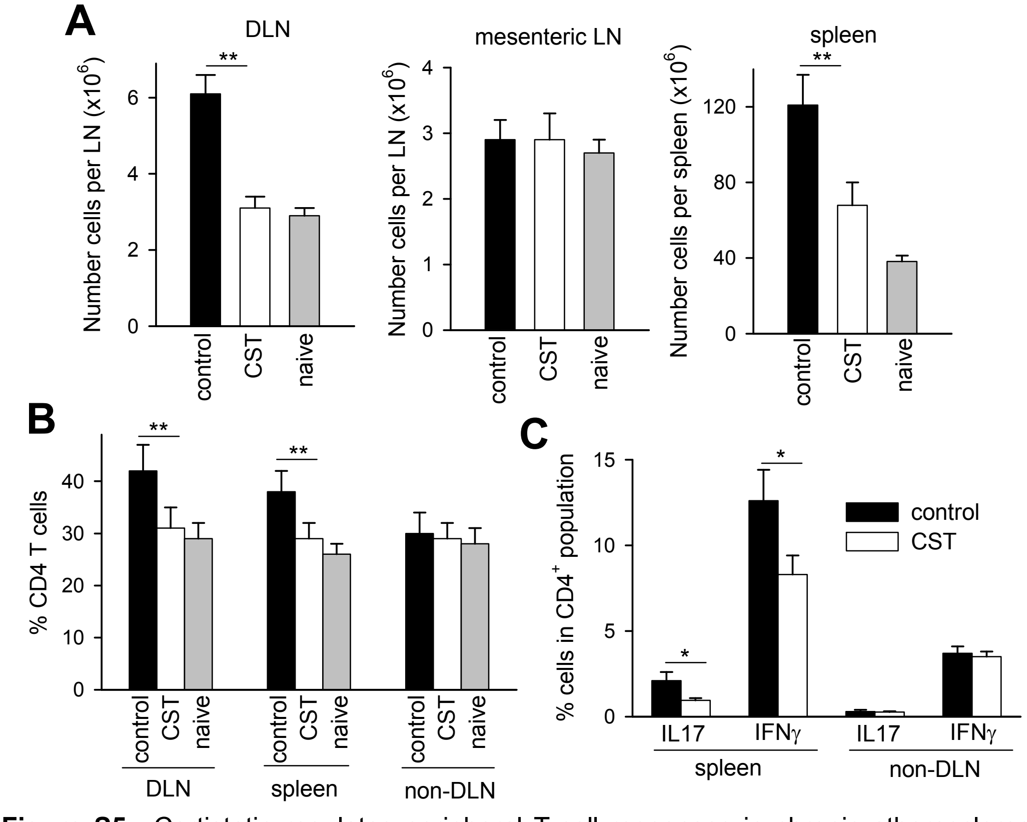

Supplementary Figure S5.- Cortistatin regulates peripheral T-cell responses in chronic atherosclerosis. ApoE$^{-/-}$ mice fed a high-fat diet during 16 weeks were treated with PBS (control) or cortistatin (CST) three times per week starting one week after initiation of diet. Naïve female C57Bl/6 (22 weeks-old) mice fed a normal diet were used as reference. **A.** Total cell numbers determined in aortic draining lymph nodes (DLN), non-draining mesenteric lymph nodes and spleens. **B.** Flow cytometric analysis of the percentage of CD4$^+$ lymphocytes in DLNs, non-draining mesenteric lymph nodes and spleens. **C.** Percentage of IFNγ- and IL-17-producing cells in the CD4 population in spleens and non-draining mesenteric lymph nodes determined by flow cytometry. n=6 mice/group, performed in two independent experiments. *p<0.05, **p<0.001 *vs* control.

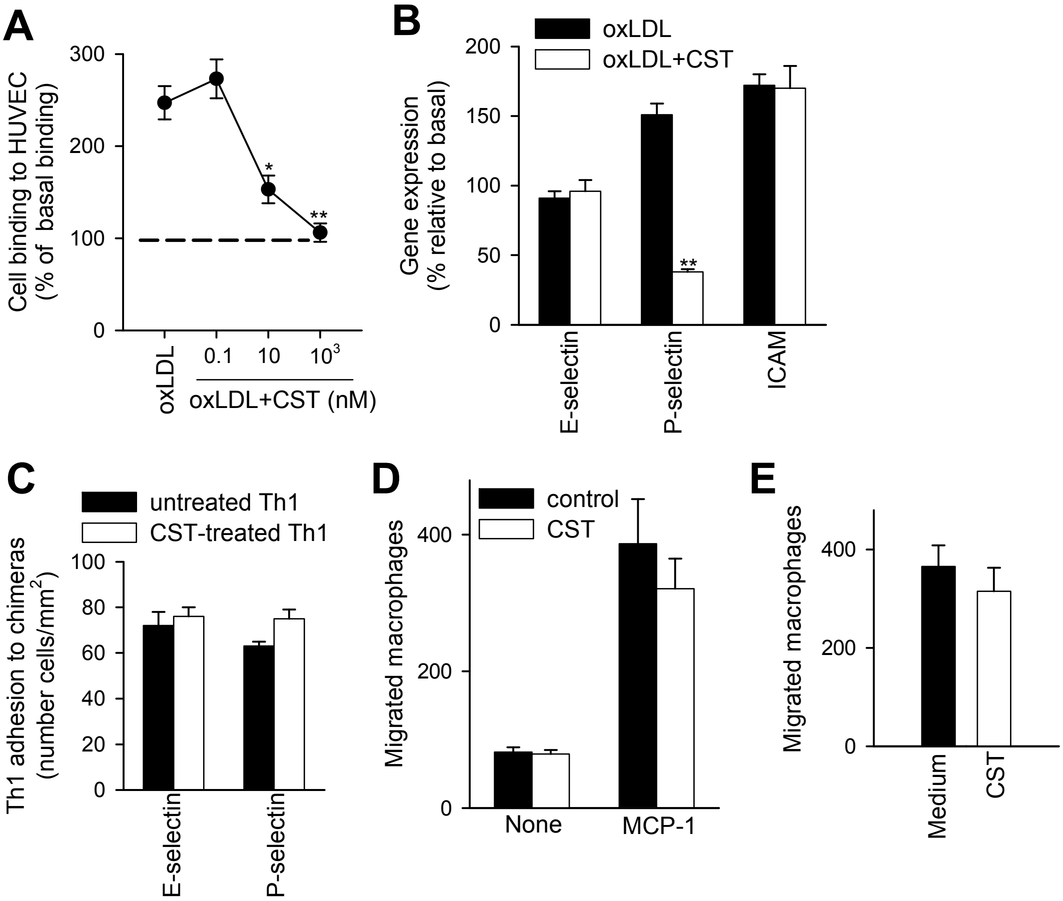

Supplementary Figure 6.- Effects of cortistatin in binding of human monocytes to activated endothelial cells and in macrophage migration. **A.** HUVEC monolayers activated with oxLDL in the absence or presence of various concentrations of cortistatin (CST) for 24 hours were co-incubated with human monocytic THP1 cells for 1 hour. After washing, we determined the number of THP1 cells that remained bound to endothelial cells. Cell binding was expressed relative to untreated oxLDL-activated HUVECs. n=4, in duplicates. *p<0.05, **p<0.01 *vs* oxLDL-activated cells. **B.** Gene expression of adhesion molecules was determined by quantitative real-time PCR in the RNA samples isolated from HUVEC monolayers activated with oxLDL in the absence or presence of 10 nM cortistatin for 24 hours. n=4, in duplicates. **p<0.01 *vs* oxLDL-activated cells. **C.** Th1 cells differentiated in the absence (untreated Th1, black columns) or presence of cortistatin (CST-treated Th1, white columns) were assayed for binding to P-selectin and E-selectin chimeras. **D.** Migration capacity against medium (none) or a chemotactic stimuli (MCP-1) of peritoneal macrophages isolated from PBS-treated (control) or cortistatin-treated apoE$^{-/-}$ mice fed a high-fat diet for 16 weeks. n=6 mice/group. **E.** Migration capacity against MCP-1 of peritoneal macrophages isolated from apoE$^{-/-}$ mice fed a high-fat diet for 16 weeks and treated *in vitro* with medium or cortistatin (100 nM). n=3 experiments, in duplicates.
